# New strategy for suppressing the growth of lung cancer cells harboring mutations in the ATP‐binding region of EGFR by targeting the molecular motor MYO1D

**DOI:** 10.1002/ctm2.515

**Published:** 2021-08-06

**Authors:** Yoo‐Seung Ko, Hyuno Kang, Jeong A Bae, Sung Jin Kim, Nacksung Kim, Ik Joo Chung, Kyung‐Sub Moon, Jin Kyung Rho, Hangun Kim, Hyung‐Ho Ha, In‐Jae Oh, Kyung Keun Kim

**Affiliations:** ^1^ Department of Pharmacology Chonnam National University Medical School Hwasun 58128 Korea; ^2^ Combinatorial Tumor Immunotherapy MRC Chonnam National University Medical School Hwasun 58128 Korea; ^3^ Division of Analytical Science Korea Basic Science Institute Daejeon 34133 Korea; ^4^ Department of Internal Medicine Chonnam National University Medical School Hwasun 58128 Korea; ^5^ Department of Neurosurgery Chonnam National University Medical School Hwasun 58128 Korea; ^6^ Department of Convergence Medicine University of Ulsan, College of Medicine Seoul 05505 Korea; ^7^ College of Pharmacy Sunchon National University Sunchon 57922 Korea

**Abbreviations**
EGFR: epidermal growth factor receptor
NSCLC: nonsmall cell lung cancer
RTK: receptor tyrosine kinase
TIRF: total internal reflection fluorescence
TKI: tyrosine kinase inhibitor

Dear Editor,

In this study, we describe that the molecular motor MYO1D holds both wild‐type and mutant EGFRs in the plasma membrane via interaction of MYO1D with a C‐terminal portion of the kinase domain (C‐terminal substrate‐binding lobe or C‐lobe) that does not contain the ATP‐binding region. Further, we show that the knockdown of MYO1D suppresses the growth, invasion and downstream signals of lung cancer and glioblastoma cells expressing mutant EGFR or mutant ErbB2, which have resistance to afatinib, osimertinib, and cetuximab. This represents a new strategy of reducing the wild‐type and mutant EGFRs themselves to treat acquired resistance in various EGFR‐overexpressing cancers harboring mutations in the ATP‐binding region, which is directly targeted by most tyrosine kinase inhibitors (TKIs).

The main challenge during EGFR‐targeted therapy in nonsmall cell lung cancer (NSCLC) is the acquisition of resistance to EGFR‐TKIs.^1^ Mechanisms of therapeutic resistance include mutations of the kinase domain at the T790M or C797S codon that interfere with TKI access to the active site, which is difficult to treat with standard therapeutic options.^2^, ^3^

The EGFR (ErbB1) and ErbB2 pathways are functionally linked and pivotal in the progression of NSCLC.^1^ Overexpression of ErbB2 has been reported in about 20% of lung cancers in which mutant ErbB2 is more potent than the wild type in promoting tumorigenicity.^4^, ^5^ Thus, combined inhibition of ErbB receptors could prevent a molecular feedback loop responsible for acquired resistance to existing anti‐ErbB agents.^6^, ^7^ To this aim, a new strategy can be considered: a single multitargeted agent and a new class of anti‐ErbB agent with a different molecular mechanism.

Previously, we provided the first evidence that the molecular motor MYO1D helps to maintain cell‐surface ErbB receptor levels (except ErbB3) by holding the receptor to the plasma membrane.^8^ Here, we evaluated whether MYO1D also holds mutant EGFRs on the NSCLC cell surface and whether blockade of MYO1D function affects the proliferation and survival of NSCLC cells with variable resistance to existing anti‐ErbB agents.

As we previously identified that the kinase domain (KD) of EGFR was needed for binding of MYO1D to EGFR in colorectal cancer (CRC) cells,^8^ we first checked whether the ATP‐binding region within the KD participates in the binding between MYO1D and EGFR in NSCLC cells. The KD is characterized by an N‐terminal ATP‐binding lobe (N‐lobe) and a C‐lobe.^9^ We found that in NSCLC cells, MYO1D interacted with the C‐lobe, which does not bear the ATP‐binding region (Figure 1A, left). We also designed a deletion construct of full‐length EGFR (Δ856‐979 EGFR; Supporting Information Figure S1A) that lacked the C‐lobe of the KD (856‐979 aa) and found that MYO1D interacted with wild‐type EGFR, but not with Δ856‐979 EGFR (Figure 1A, middle). This finding indicated that MYO1D interacts with EGFR through the C‐lobe of the KD and that the ATP‐binding region within the KD is not involved in the interaction of MYO1D with EGFR in NSCLC cells. That is, the molecular motor MYO1D also holds mutant EGFRs in the plasma membrane irrespective of mutations in the ATP‐binding region (N‐lobe).

**FIGURE 1:**
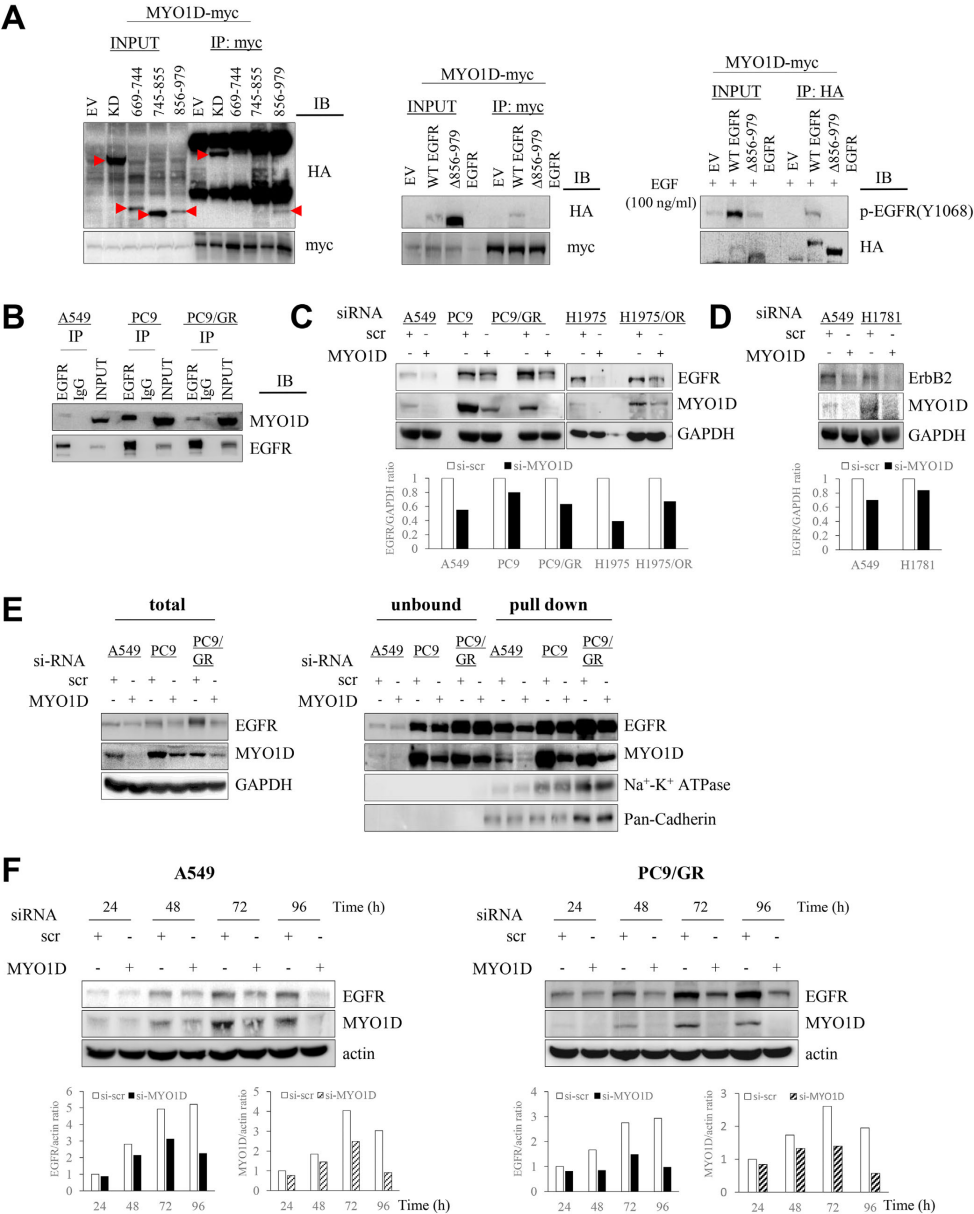
MYO1D retains EGFR in the plasma membrane in NSCLC cells expressing either wild‐type or mutant EGFR. A, MYO1D interacts with EGFR through the C‐lobe of the kinase domain (KD) and the ATP‐binding region within the KD is not involved in the interaction of MYO1D with EGFR. PC9/GR cells were transfected with empty vector (EV), HA‐tagged EGFR KD, or each HA‐tagged deletion mutant of EGFR KD (left), or with EV, HA‐tagged full‐length EGFR (WT EGFR), or HA‐tagged EGFR deletion construct (Δ856‐979 EGFR) lacking the C‐lobe of the KD (middle, right) for 48 h. KD was included as a positive control for the interaction of MYO1D with EGFR (left). Cell lysates were immunoprecipitated with the MYO1D antibody and analyzed by immunoblotting with an HA antibody (middle) or were immunoprecipitated with an HA antibody and analyzed by immunoblotting with a phospho‐EGFR antibody (right). The molecular characteristic of C‐lobe‐deleted EGFR was confirmed by phosphorylation status after treatment with EGF (right). The C‐lobe‐deleted EGFR was not phosphorylated after treatment with EGF for 30 min because of the inhibited activation of EGFR owing to the incomplete formation of an asymmetric N‐lobe/C‐lobe KD dimer. B, MYO1D interacts directly with both endogenous mutant and wild‐type EGFR. A549, PC9, or PC9/GR NSCLC cell lysates were immunoprecipitated with an EGFR antibody and immunoblotted with the indicated antibodies. C, Knockdown of MYO1D reduces the expression level of EGFRs. A549, PC9, PC9/GR, H1975, and H1975/OR cells were transfected with scrambled si‐RNA (si‐scr) or si‐RNAs targeting MYO1D (si‐MYO1D, mixed with si‐MYO1D #1 and another independent si‐MYO1D #2, see Materials and Methods) for 48 h. After transfection, cells lysates were analyzed with the indicated antibodies. D, Knockdown of MYO1D reduces the expression level of mutant ErbB2 in H1781 cells. H1781 cells were transfected with either si‐scr or si‐MYO1D for 48 h. After transfection, cells were analyzed by immunoblotting with the indicated antibodies. A549 cells were included as a control. E, Knockdown of MYO1D inhibits the expression level of EGFRs in the membrane fraction. A549, PC9, or PC9/GR cells were transfected with either si‐scr or si‐MYO1D for 48 h and were biotinylated. The plasma membrane preparations made by using immobilized avidin (pull down) were subjected to analysis with the indicated antibodies. The obtained subcellular fraction was confirmed with anti‐Na^+^‐K^+^‐ATPase antibody and anti‐Pan‐Cadherin antibody. F, The delay between EGFR downregulation and knockdown of MYO1D. EGFR levels are shown after the time course of knockdown of MYO1D in A549 or PC9/GR cells. Cells were treated with either scrambled (si‐scr) or MYO1D‐specific si‐RNA (mixed si‐MYO1D #1 with si‐MYO1D #3, see Materials and Methods) and analyzed for the expression of EGFR and MYO1D at the indicated times after transfection

As most EGFR mutations occur in the ATP‐binding region,^1^, ^2^ we thus examined the interactions of MYO1D with EGFRs in both wild‐type and mutant NSCLC cells, such as *EGFR* wild‐type A549 cells, gefitinib‐sensitive PC9 cells harboring an *EGFR* exon 19 deletion, and gefitinib‐resistant PC9/GR cells harboring an *EGFR* exon 19 deletion/T790M. We found that MYO1D interacted with EGFRs in all three cell lines (Figure 1B). Next, we investigated the expression levels of wild‐type and mutant EGFR after knockdown of MYO1D with si‐MYO1D. Knockdown of MYO1D reduced the level of EGFR in A549, PC9, and PC9/GR cells (Figure 1C) and in HCC827 and NCI‐H1650 cells harboring an *EGFR* exon 19 deletion (Supporting Information Figure S1B). Also, EGFR was reduced after knockdown of MYO1D in osimertinib‐sensitive H1975 cells harboring L858R/T790M, and osimertinib‐resistant H1975/OR cells (Figure 1C), which acquired resistance to osimertinib through the epithelial‐to‐mesenchymal transition.^10^ Moreover, ErbB2 expression was decreased after knockdown of MYO1D in NCI‐H1781 lung cancer cells (Figure 1D) expressing wild‐type EGFR and mutant ErbB2 containing a Val‐Cys insertion at G776 in exon 20 of the ErbB2 gene. Furthermore, in NSCLC cells treated with si‐MYO1D, the level of EGFR was decreased in membrane preparations from MYO1D‐depleted A549, PC9, and PC9/GR cells (Figure 1E). To exclude the possibility of off‐target effects, we performed a rescue experiment in which MYO1D was overexpressed in the presence of si‐MYO1D. The decreased localization of EGFR at the membrane and the resultant cell viability and motility after treatment with si‐MYO1D were restored by expression of exogenous MYO1D in A549 and PC9/GR cells (Supporting Information Figure S2), indicating the specificity of targeting MYO1D. We examined the time course of decreasing levels of EGFR after knockdown of MYO1D in A549 and PC9/GR cells and found that the EGFR level was substantially decreased at 48 h and persisted for up to 96 h after knockdown of MYO1D (Figure 1F), indicating a delay between EGFR downregulation and knockdown of MYO1D.

Confocal images revealed that the cell surface localization of EGFR was impaired in MYO1D‐depleted NSCLC cells (Figure 2A). We detected MYO1D and EGFR molecules at the plasma membrane of A549 and PC9/GR cells by using total internal reflection fluorescence (TIRF) microscopy (Figure 2B, left). About one‐half of the MYO1D population was colocalized with EGFR regardless of si‐RNA treatment or cell types. EGFR and colocalized EGFR with MYO1D were decreased significantly by 23% and 48% in si‐MYO1D‐treated A549 cells and by 24% and 69% in si‐MYO1D‐treated PC9/GR cells, respectively, compared with those of the control group (Figure 2B, right). Overall, MYO1D knockdown downregulates the interaction between MYO1D and EGFR in the plasma membrane and thereby subsequently induces EGFR disengagement from the plasma membrane of NSCLC cells.

**FIGURE 2:**
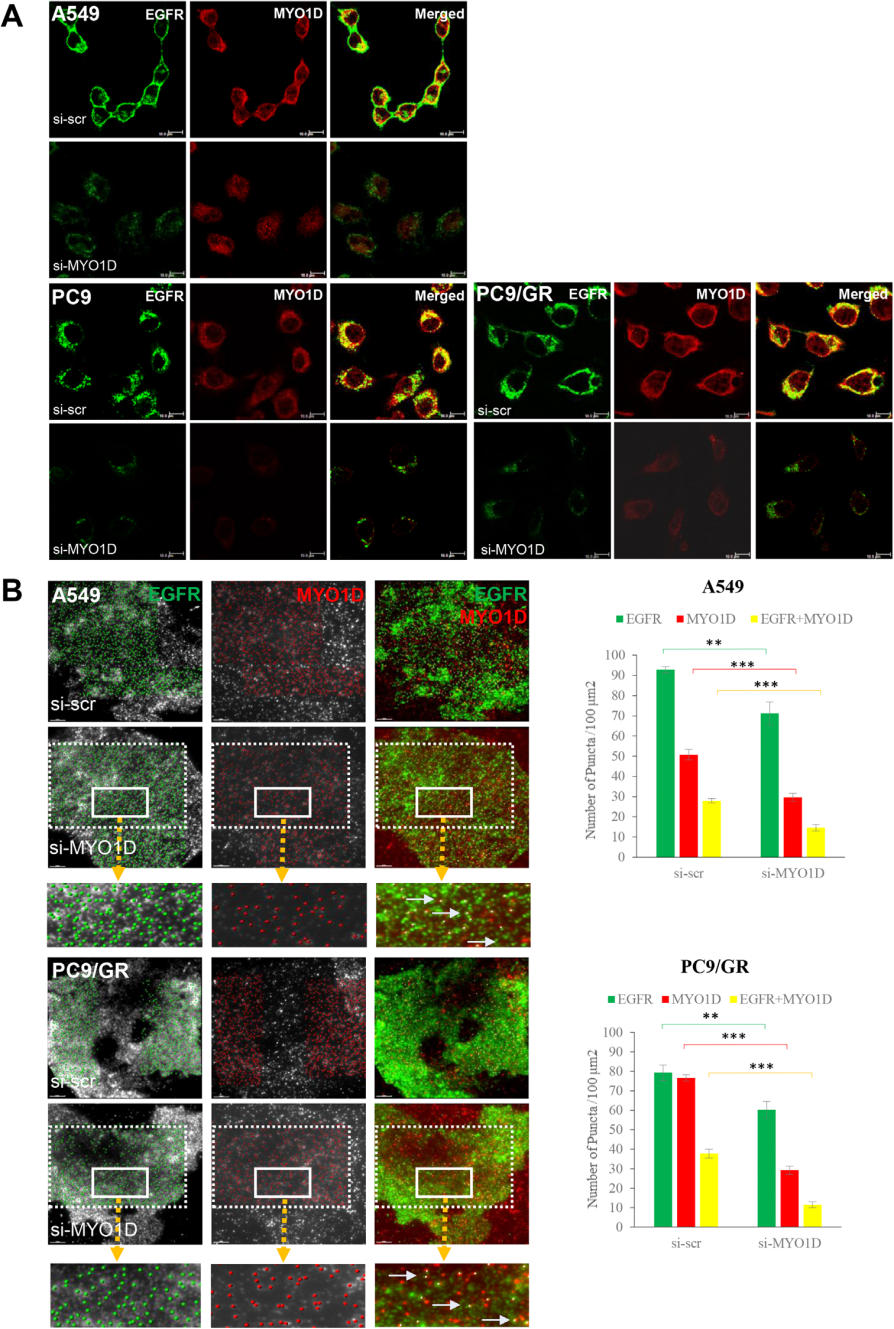
The cell surface expression of mutant EGFRs and colocalized EGFR‐MYO1D clusters are reduced in MYO1D‐depleted NSCLC cells. A, Confocal images of expression of EGFRs and MYO1D in MYO1D‐depleted NSCLC cells. The cell surface expressions of wild‐type and mutant EGFRs are reduced in MYO1D‐depleted NSCLC cells. A549 (upper), PC9 (middle), and PC9/GR (lower) cells were transfected with either si‐scr or si‐MYO1D and stained with the indicated antibodies, and then the confocal images were obtained. EGFR level (green) was markedly diminished at the surface of MYO1D (red)‐depleted cells. Scale bar: 10 μm. B, Number of EGFR puncta and colocalized EGFR‐MYO1D clusters were significantly decreased when MYO1D was knocked down in both A594 and PC9/GR cells. Locations of EGFR and MYO1D molecules were visualized with a TIRF microscope and marked with green and red dots, respectively, on the gray‐scale TIRF images. Boxes with a dashed line represent the area where fluorescent puncta were counted on that specific image. Areas in the solid lined boxes are enlarged at the bottom for a better view, and the arrows indicate colocalized puncta in yellow. The histogram shows the number of puncta per 100 μm^2^ of cell surface counted with Imaris (mean ± SEM, *n* = 6 or 7). Scale bar = 10 μm. The asterisk represents a significant difference between groups (**P < .01; ***P < .001)

To discern whether MYO1D contributes to regulating the cell survival and motility of NSCLC cells expressing wild‐type or mutant EGFR, we analyzed cell proliferation and motility in A549, PC9, PC9/GR, HCC827, NCI‐H1650, H1975, H1975/OR, and H1781 cells. Knockdown of MYO1D attenuated the proliferation of NSCLC cells expressing either wild‐type or mutant EGFR, or ErbB2 (Figures 3A and S1C). Next, we compared the effect of MYO1D depletion with those of EGFR‐targeted TKIs on cell survival and motility in cells treated with gefitinib, afatinib, or osimertinib. Cell proliferation and invasion were significantly inhibited in PC9 cells treated with gefitinib or afatinib from a concentration of 0.01 μM, whereas the two parameters were somewhat resistant to treatment with these drugs in A549 and PC9/GR cells (Figures 3B and 3E and Supporting Information Figures S3A and S3B). On the other hand, afatinib and osimertinib showed inhibitory effects in H1975 cells from a concentration of 1 μM, whereas H1975/OR cells were resistant to both drugs (Figure 3B). Interestingly, MYO1D knockdown reduced the cell proliferation of PC9/GR cells by 40% at 48 h after transfection and by 35% at 72 h after transfection in H1975/OR cells, and these inhibitory effects were superior to those of afatinib (14% at 0.01 μM) and osimertinib (6% at 0.1 μM) (Figure 3C). Cell invasion was significantly reduced in all NSCLC cell lines tested when they were treated with si‐MYO1D (Figure 3D and Supporting Information Figure S1D). The clonogenic assay further demonstrated that knockdown of MYO1D reduced the cell survival rate of NSCLC cells expressing either wild‐type or mutant EGFR (Supporting Information Figure S3D) compared with those by gefitinib or afatinib, which showed varying effectiveness among NSCLC cells (Supporting Information Figures S3C and S3E). We found that MYO1D‐depleted H1781 cells showed decreased cell proliferation (Figure 3A), invasion (Figure 3D), and survival (Supporting Information Figure S3D), whereas these measures were not significantly altered in afatinib‐treated cells (Figure 3B and 3E, and Supporting Information S3E). These results indicated that the downregulation of cell viability and motility in si‐MYO1D‐treated cells was derived from the decreased plasma membrane levels of wild‐type and mutant EGFR or mutant ErbB2 after knockdown of MYO1D.

**FIGURE 3:**
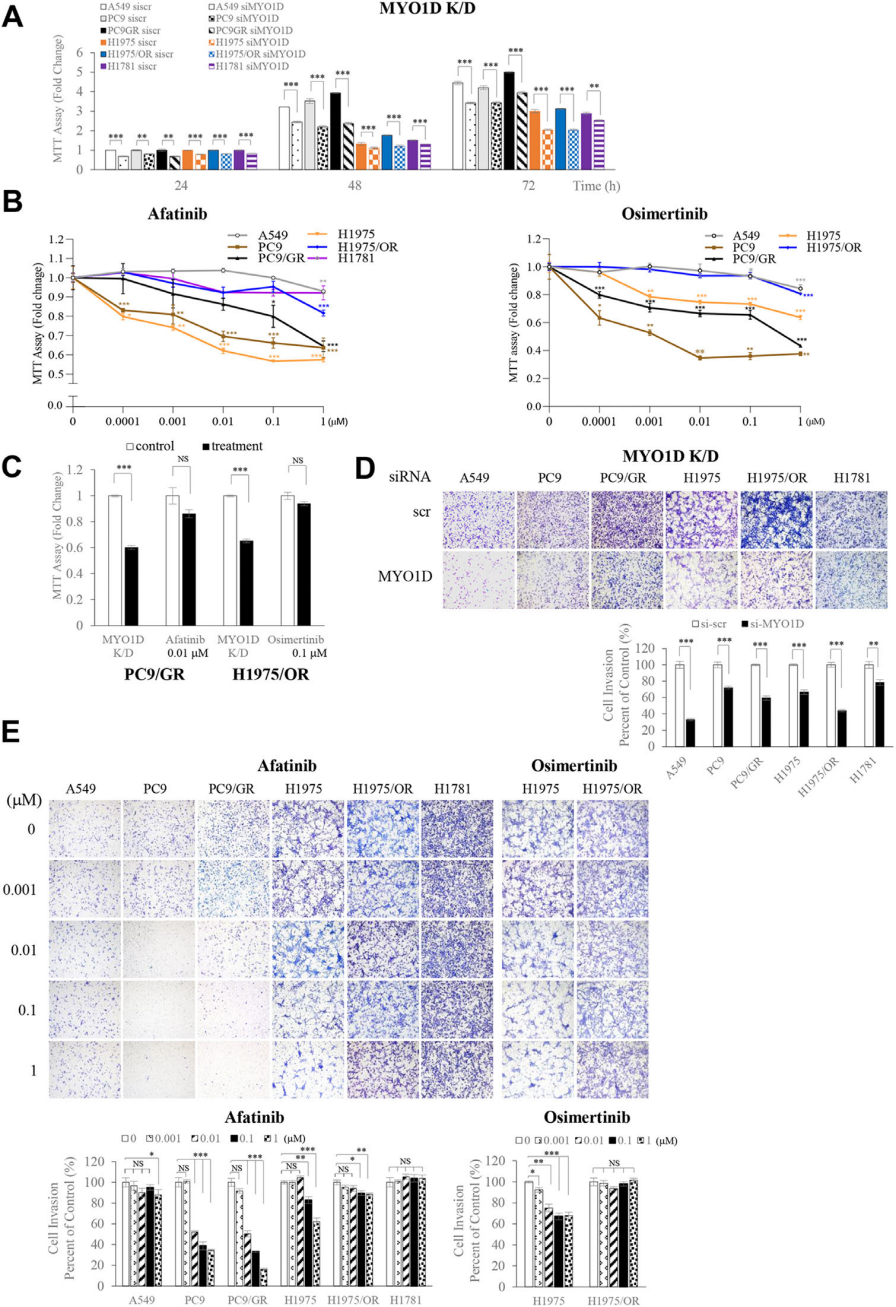
Depletion of MYO1D reduces cell proliferation, motility, and survival in NSCLC cells expressing either wild‐type or mutant EGFR, or mutant ErbB2. A, Knockdown of MYO1D suppresses the proliferation of NSCLC cells. Cell proliferation was measured by MTT assays at 24, 48, and 72 h posttransfection of si‐scr or si‐MYO1D in A549, PC9, PC9/GR, H1975, H1975/OR, or H1781 cells. B, NSCLC cells expressing either wild‐type or mutant EGFR, or mutant ErbB2 show varying sensitivity to existing TKIs. These were summarized as dose curves. A549, PC9, PC/9GR, or H1781 cells were treated with afatinib or osimertinib for 48 h, and cell proliferation was measured by MTT assay. H1975 or H1975/OR cells were treated with afatinib or osimertinib for 48 h and subjected to MTT assay. C, Comparison of effects on cell proliferation between MYO1D‐knockdown and afatinib in PC9/GR cells or osimertinib in H1975/OR cells. D, Knockdown of MYO1D suppresses the invasion capacity of NSCLC cells expressing either wild‐type or mutant EGFR, or mutant ErbB2. A549, PC9, PC9/GR, H1975, H1975/OR, or H1781 cells were transfected with either si‐scr or si‐MYO1D for 48 or 72 h and subjected to transwell invasion assay. The histogram reveals invading cells, which were quantified at the five random areas, and the asterisk represents a significant difference between groups (**P < .01; ***P < .001). E, Effect of various TKIs on cell invasiveness in NSCLC cells expressing either wild‐type or mutant EGFR, or mutant ErbB2. The invasion capacity of cells following treatment with the indicated concentrations of afatinib or osimertinib for 24 h was determined by using the transwell invasion assay. The histogram and pictures of the cell invasion analysis were obtained as in Figure 3D

Degradation of EGFR is mediated by E3 ubiquitin ligase, Cbl‐b, and/or c‐Cbl.^11^ We previously showed that the reduced ErbB4 by knockdown of MYO1D correlates with proteasomal degradation.^8^ We thus checked whether the effect of MYO1D knockdown on the decrease in EGFR in PC9/GR cells was modified by depletion of Cbl‐b or c‐Cbl. The decreased EGFR after MYO1D knockdown was restored by pretreatment with si‐Cbl‐b or si‐c‐Cbl in PC9/GR cells (Figure 4A and Supporting Information Figure S4A). To prove the effectiveness of Cbl E3 ligase in regulating EGFR, we further analyzed the cellular phenotype in MYO1D‐knockdown cells. The decreased invasion after MYO1D knockdown was partially rescued by pretreatment with si‐Cbl‐b or si‐c‐Cbl in PC9/GR cells (Figure 4B and Supporting Information Figure S4B), and c‐Cbl‐induced ubiquitination of EGFR was markedly increased by depletion of MYO1D (Figure 4C). EGFR interacted with Cbl‐b and c‐Cbl in PC9/GR cells; however, the interaction was greater in the si‐MYO1D‐treated cells (Figure 4D). In MYO1D‐knockdown NSCLC cells, EGFR downstream signals, such as p‐AKT, p‐ERK, and p‐p38, which are associated with cell proliferation and migration, were reduced (Figure 4E).

FIGURE 4EGFRs disengaged from the plasma membrane after knockdown of MYO1D are degraded in a Cbl E3‐ligase‐dependent manner. A, The reduced EGFR level in MYO1D‐depleted PC9/GR cells was restored by knockdown of Cbl‐b or c‐Cbl. PC9/GR cells were depleted with si‐RNA targeting Cbl‐b (#1, left) or c‐Cbl (#1, right) for 48 h. After transfection, cells were immunoblotted with the indicated antibodies. B, Reduced invasiveness in MYO1D‐depleted PC9/GR cells is rescued by depletion of Cbl‐b or c‐Cbl. The histogram and pictures of the cell invasion analysis were obtained as in Figure 3D. C, c‐Cbl associates with ubiquitination of EGFR in MYO1D‐depleted PC9/GR cells. PC9/GR cells were transfected with either si‐scr or si‐MYO1D and c‐Cbl or ubiquitin. The cell products immunoprecipitated with an EGFR antibody were separated on 6% SDS‐polyacrylamide gels, transferred, and immunoblotted with an antiubiquitin antibody. D, The interaction of EGFR with Cbl‐b or c‐Cbl is increased in MYO1D‐depleted cells. PC9/GR cells were transfected with either si‐scr or si‐MYO1D for 48 h, and lysates were immunoprecipitated with an EGFR antibody and analyzed by immunoblotting with the indicated antibodies. E, Knockdown of MYO1D inhibits downstream signals of EGFR. A549, PC9, or PC9/GR cells were transfected with either si‐scr or si‐MYO1D for 48 h, and lysates were immunoblotted with the indicated antibodies. F, Schematic showing that MYO1D holds both mutant and wild‐type EGFRs in the plasma membrane of NSCLC and glioblastoma cells. As in wild‐type EGFR, MYO1D also interacted with the C‐lobe of the kinase domain of mutant EGFRs, which are responsible for resistance to existing anti‐EGFR agents and allowed them to activate their downstream signals for cell proliferation, motility, and survival. After knockdown of MYO1D, both wild‐type and mutant EGFRs are released from MYO1D that is anchored on the underlying actin cytoskeleton and the disengaged EGFRs are downregulated in the cytosol through a Cbl E3‐ligase‐dependent proteasomal degradation

**Figure:**
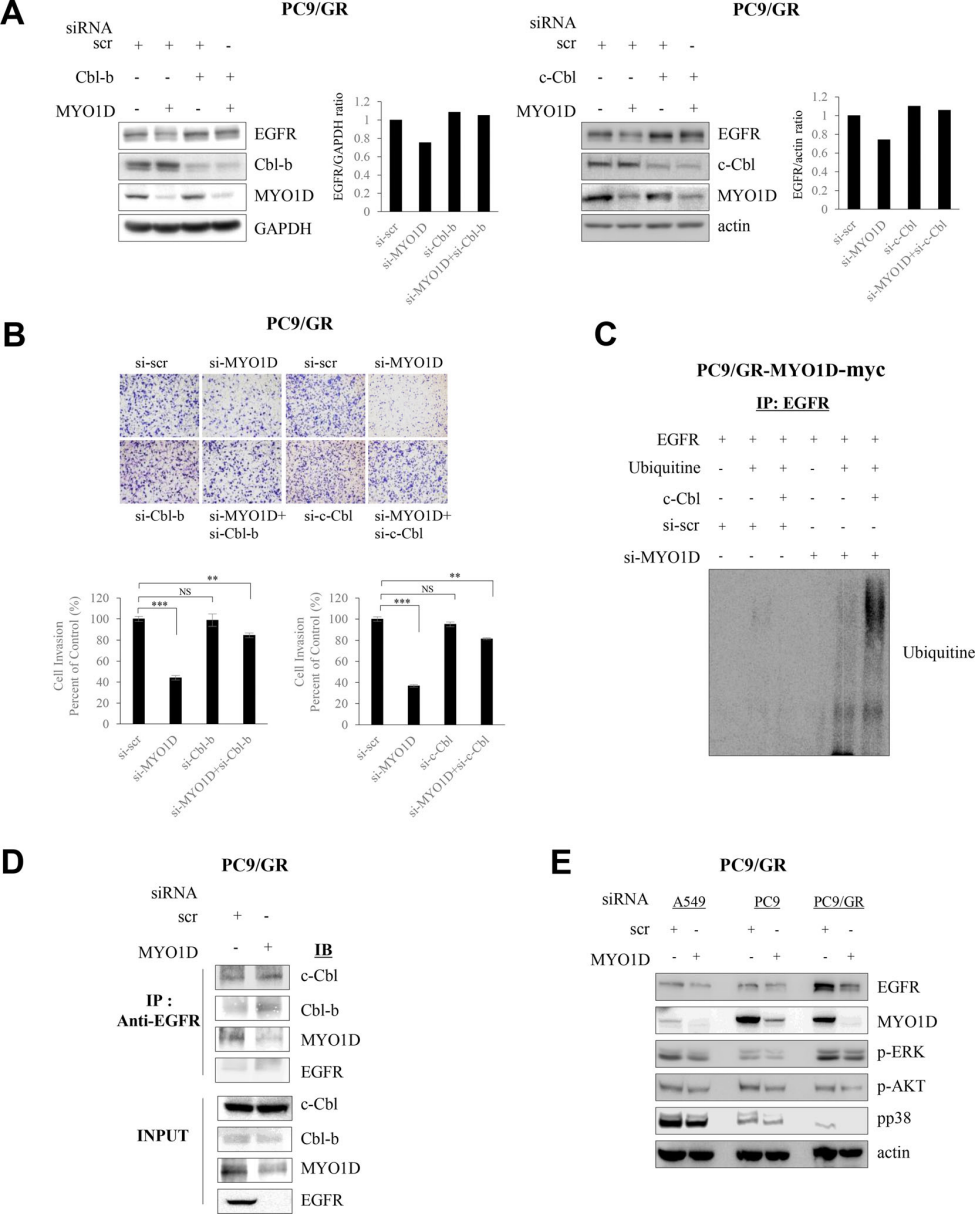

**Figure:**
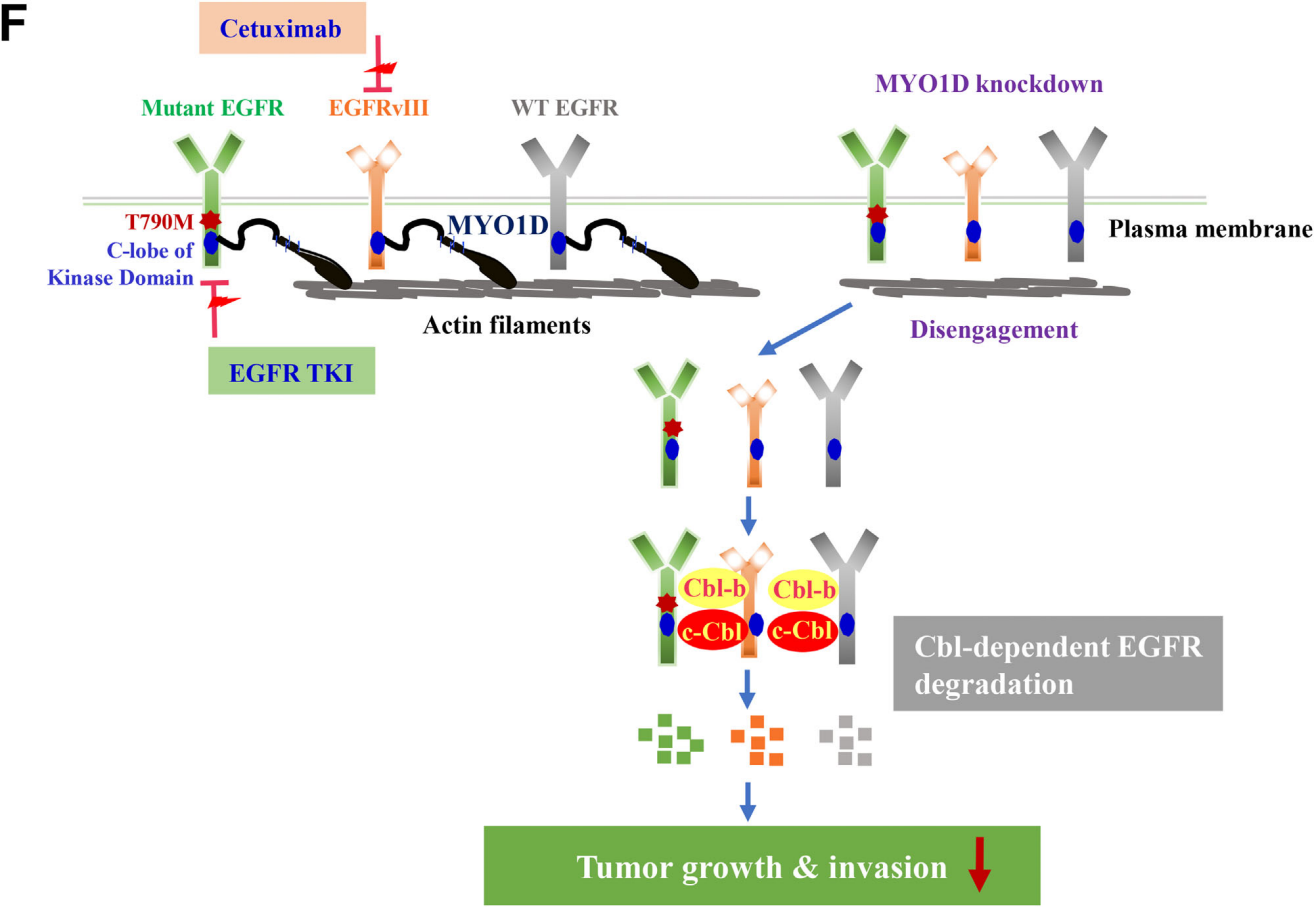

The *EGFR* gene is overexpressed in about one‐half of glioblastoma patients, which is accompanied by various EGFR mutants, of which the EGFRvIII mutant is common.^7^ To confirm whether MYO1D also regulates wild‐type and mutant EGFRs in glioblastoma cells, we examined the in vitro phenotypes in U87‐MG glioblastoma (U87) cells. MYO1D interacted with EGFR in both wild‐type and mutant EGFRvIII‐expressing U87 cells (Supporting Information Figure S5A). However, MYO1D did not interact with mutant EGFR with Δ‐kinase, which lacks a kinase domain. After knockdown of MYO1D, the level of EGFR was decreased in U87 cells expressing either wild‐type EGFR or mutant EGFRvIII, except in cells expressing the mutant EGFR with Δ‐kinase (Supporting Information Figure S5B). Also, knockdown of MYO1D reduced cell invasion (Supporting Information Figure S5C), survival (Supporting Information Figure S5D), and proliferation (Supporting Information Figure S5E) in both cell lines. Afatinib did not affect the cell proliferation (Supporting Information Figure S5F) or survival (Supporting Information Figure S5G) of either glioblastoma cell line. Furthermore, cetuximab, an EGFR monoclonal antibody, did not inhibit the cell invasion or proliferation of either glioblastoma cell line but inhibited the invasiveness of Caco2 CRC cells (Supporting Information Figure S5H). Moreover, MYO1D knockdown reduced EGFR downstream signals in the U87 cells, such as p‐Stat‐3 and p‐AKT (Supporting Information Figure S5I).

These results indicated that, in addition to NSCLC cells harboring mutated EGFR in the cytoplasmic ATP‐binding domain, MYO1D knockdown decreased the cell proliferation and invasiveness of glioblastoma cells harboring mutated EGFR in the extracellular domain through a Cbl E3‐ligase‐dependent proteasomal degradation of EGFR (Figure 4F).

In conclusion, our findings provide an experimental rationale for developing novel therapeutics in lung cancer and glioblastoma patients harboring various *EGFR* gene mutations via reduction of the wild‐type and mutant EGFRs themselves before they are activated after binding with their corresponding ligands. Whereas EGFR‐TKIs suppress the growth of cancer cells harboring activating mutations of EGFRs after they bind with their corresponding ligands, targeting MYO1D affects the growth of EGFR‐mutant cancer cells before they can bind with their corresponding ligands. Thus, our present findings suggest that the combined regimen of TKI and a new class of anti‐EGFR agent, a blocker of MYO1D function, is an attractive strategy for treating acquired resistance to EGFR‐TKIs, including osimertinib‐resistant NSCLC.

## CONFLICT OF INTEREST

The authors declare that there is no conflict of interest.

## ETHICS STATEMENT

All animal experiments were performed under the guidelines of the Chonnam National University Medical School Research Institutional Animal Care Committee, and all the experimental protocols were approved by the committee (CNU IACUC‐H‐2020‐12).

## AUTHOR CONTRIBUTIONS

K.K.K., O.I.J. conceptualization and writing‐original draft; K.Y.S., K.H., B.J.A., K.S.J. investigation; K.N., C.I.J., M.K.S., R.J.K., K.H., H.H.H. supervision.

## DATA AVAILABILITY STATEMENT

All the data generated or analyzed during this study are included in this published article and its additional supporting files.

## Supporting information


**FIGURE S1** Map of deletion constructs of EGFR and the effects of MYO1D‐knockdown on cell proliferation and motility of HCC827 and NCI‐H1650 NSCLC cells. A, Schematic diagram showing the three deletion mutants in the kinase domain (KD) of EGFR and the EGFR deletion construct (Δ856‐979 EGFR) lacking the C‐lobe of the KD. ATP‐BR, ATP‐binding region; ECD, extracellular domain; CTD, cytoplasmic domain. B–D, Knockdown of MYO1D reduced the EGFR level (B) and suppressed the cell proliferation (C) and invasiveness (D) of NSCLC cells. HCC827 and NCI‐H1650 cells were transfected with either si‐scr or si‐MYO1D for 24, 48, or 72 h and subjected to immunoblot assay, MTT assay, or transwell invasion assay. The histograms of the cell proliferation and invasion analyses were obtained as in Figure 3A and  3D, respectively.

**FIGURE S2** Expression of exogenous MYO1D restores the decreases in the EGFR level, cell proliferation, and motility caused by treatment with MYO1D si‐RNA in NSCLC cells. Rescue experiments were performed by transfection with MYO1D siRNAs (siRNA#1 and siRNA#2) overnight followed by transfection of the plasmid, a construct expressing myc‐tagged MYO1D (MYO1D‐myc). A, Reduced EGFR level was rescued by the expression of exogenous MYO1D in the membrane fraction. MYO1D was overexpressed in A549 cells in the presence of siRNA targeting MYO1D. Plasma membrane preparations were obtained as in Figure 1E. B, Expression of exogenous MYO1D attenuated the suppression of cell proliferation by MYO1D depletion in A549 cells. C, Exogenous MYO1D protein restored the decrease in invasion capacity by MYO1D depletion in A549 cells. The histogram and pictures of the cell invasion analysis were obtained as in Figure 3D. D, Exogenous MYO1D protein rescued the EGFR level of PC9/GR cells in the membrane fraction after MYO1D depletion. E, The suppression of cell proliferation by MYO1D depletion was attenuated by exogenous MYO1D protein in PC9/GR cells. F, The decreased invasion capacity in PC9/GR cells caused by knockdown of MYO1D was restored by exogenous expression of MYO1D protein.

**FIGURE S3** Knockdown of MYO1D reduces long‐term cell survival in NSCLC cells expressing either wild‐type or mutant EGFR, which show varying sensitivity to TKIs. A, NSCLC cells expressing either wild‐type or mutant EGFR show varying sensitivity to gefitinib. A549, PC9, or PC/9GR cells were treated with gefitinib for 48 h and cell proliferation was determined by MTT assay. B, Effect of gefitinib on cell invasiveness in NSCLC cells expressing either wild‐type or mutant EGFR. The invasion capacity of cells after treatment with the indicated concentrations of gefitinib for 24 h was determined by using the transwell invasion assay. The histogram and pictures of the cell invasion analysis were obtained as in Figure 3D. C, Effect of gefitinib on survival of NSCLC cells. Long‐term cell survival was determined by clonogenic assay after treatment with gefitinib in A549, PC9, or PC9/GR cells. After incubation for 14 days, staining of attached cells was performed with trypan blue solution. D, Knockdown of MYO1D suppresses the survival of NSCLC cells. Long‐term cell survival was determined by clonogenic assay after MYO1D depletion. A549, PC9, PC9/GR, H1975, H1975/OR, or H1781 cells were transfected with either si‐scr or si‐MYO1D. After incubation for 14 days, staining of attached cells was performed with trypan blue solution. E, Effect of various TKIs on survival of NSCLC cells. Long‐term cell survival was determined by clonogenic assay after treatment with afatinib or osimertinib in A549, PC9, or PC9/GR, H1781, H1975, or H1975/OR cells. After incubation for 14 days, attached cells were stained with trypan blue solution.

**FIGURE S4** Another set of specific si‐RNAs for Cbl‐b or c‐Cbl also restores the decreased EGFR level and invasion capacity in MYO1D‐depleted PC9/GR cells. A, Knockdown of Cbl‐b (left, mixed si‐Cbl‐b #1 with #2, see Materials and Methods) or c‐Cbl (right, mixed si‐c‐Cbl #1 with #2, see Materials and Methods) restored the reduced EGFR level in MYO1D‐depleted PC9/GR cells. After transfection, cells were immunoblotted with the indicated antibodies. B, Depletion of Cbl‐b or c‐Cbl restored the reduced invasion capacity in MYO1D‐depleted PC9/GR cells. The histogram and pictures of the cell invasion analysis were obtained as in Figure 3D.

**FIGURE S5** MYO1D retains the wild‐type EGFR and mutant EGFRvIII in the plasma membrane of U87 glioblastoma cells. A, MYO1D interacts directly with endogenous mutant EGFRvIII and wild‐type EGFR. U87 glioblastoma cells were transfected with each deletion mutant of EGFR, such as empty vector (vsvg), kinase‐domain deleted EGFR construct (Δ‐kinase), or exon 2–7 deleted EGFR construct (EGFRvIII, in‐frame 801 nucleotides‐deleted in the extracellular domain). Each cell lysate was immunoprecipitated with an EGFR antibody and analyzed by immunoblotting with the indicated antibodies. B, Knockdown of MYO1D inhibits the expression level of wild‐type and mutant EGFRs in glioblastoma cells. U87 cells were cotransfected with each deletion mutant of EGFR and si‐scr or si‐MYO1D for 48 h. After transfection, cells were immunoblotted with the indicated antibodies. C, Depletion of MYO1D suppresses the invasion capacity of glioblastoma cells expressing wild‐type EGFR or mutant EGFRvIII. U87 cells transfected with either si‐scr or si‐MYO1D for 48 h were subjected to transwell invasion assay. The histogram of the invasion assay was obtained as in Figure 3D. D, Knockdown of MYO1D suppresses the survival of glioblastoma cells expressing wild‐type EGFR or mutant EGFRvIII. U87 cells were transfected with either si‐scr or si‐MYO1D and long‐term cell survival was determined by clonogenic assay as in Supporting Information Figure S3D. E, Knockdown of MYO1D suppresses proliferation in glioblastoma cells expressing wild‐type EGFR or mutant EGFRvIII. Cell proliferation was determined by MTT assays at 24, 48, and 72 h after cotransfection with each deletion mutant of EGFR and si‐scr or si‐MYO1D. F, The proliferation of glioblastoma cells was little affected by afatinib. PC9, PC/9GR, U87/wild‐type EGFR, or U87/EGFRvIII cells were treated with afatinib for 48 h and sensitivity was determined by MTT assay. G, The survival of glioblastoma cells was also little affected by afatinib. Long‐term cell survival was determined by clonogenic assay after treatment with afatinib in glioblastoma cells as in Supporting Information Figure S3E. H, The invasiveness and proliferation of glioblastoma cells expressing mutant EGFRvIII was little affected by cetuximab, an EGFR monoclonal antibody interacting with the extracellular EGF‐binding site of EGFR. Cells treated with the indicated concentrations of cetuximab for 48 h were subjected to transwell invasion assay (upper) or MTT assay (lower). I, Knockdown of MYO1D inhibits downstream signals of EGFR in glioblastoma cells expressing wild‐type EGFR or mutant EGFRvIII. U87 cells were cotransfected with each deletion mutant of EGFR and si‐scr or si‐MYO1D for 48h, and cell lysates were immunoblotted with the indicated antibodies.

Supporting Information
