# Supplementary figures and images for: New strategy for suppressing the growth of lung cancer cells harboring mutations in the ATP‐binding region of EGFR by targeting the molecular motor MYO1D
Source: Clin Transl Med. 2021 Aug 6;11(8):e515. doi: 10.1002/ctm2.515 (PMC8343539; doi:10.1002/ctm2.515)

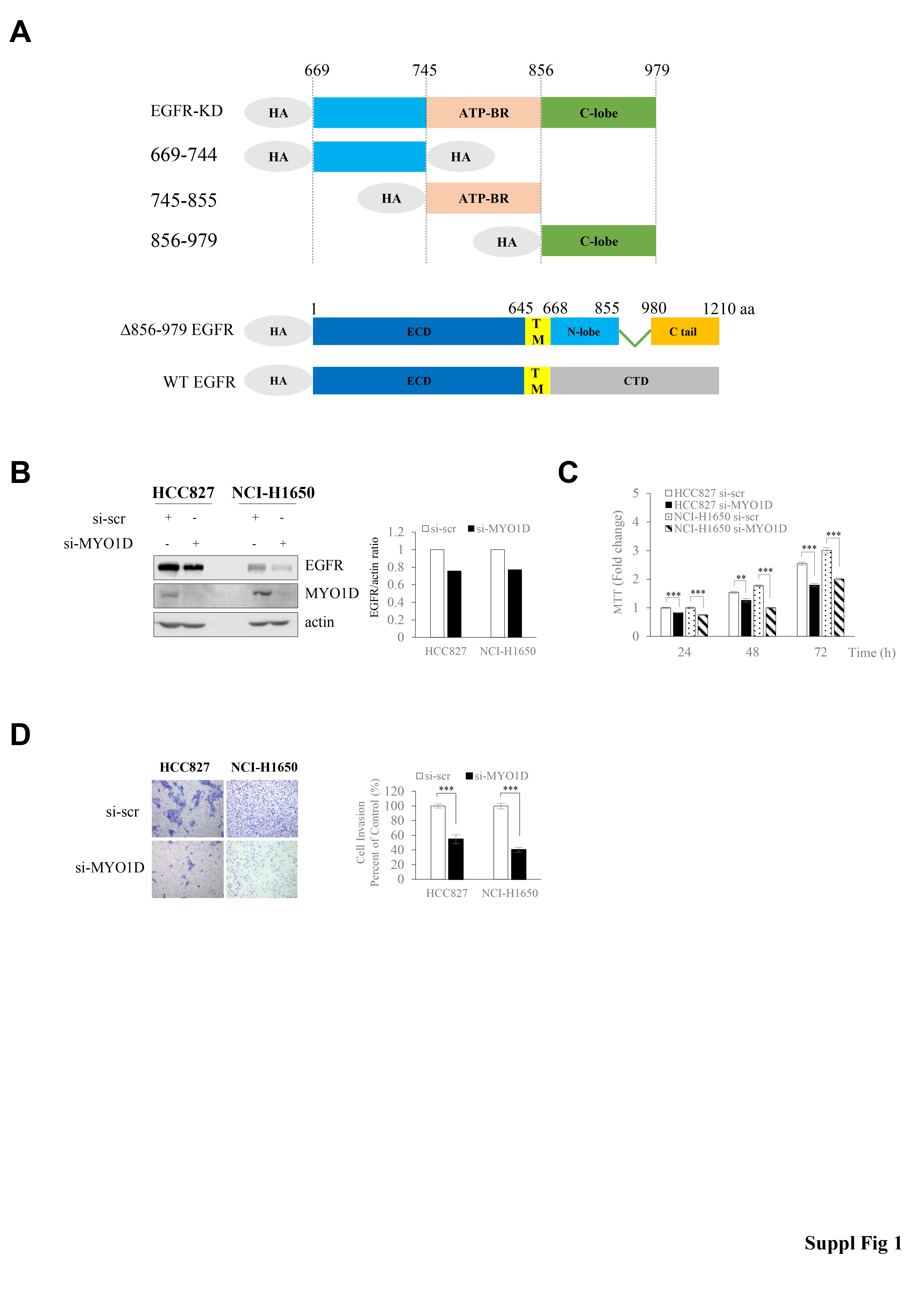

Supplement: Supplementary file 1 — FIGURE S1 Map of deletion constructs of EGFR and the effects of MYO1D‐knockdown on cell proliferation and motility of HCC827 and NCI‐H1650 NSCLC cells. A, Schematic diagram showing the three deletion mutants in the kinase domain (KD) of EGFR and the EGFR deletion construct (Δ856‐979 EGFR) lacking the C‐lobe of the KD. ATP‐BR, ATP‐binding region; ECD, extracellular domain; CTD, cytoplasmic domain. B–D, Knockdown of MYO1D reduced the EGFR level (B) and suppressed the cell proliferation (C) and invasiveness (D) of NSCLC cells. HCC827 and NCI‐H1650 cells were transfected with either si‐scr or si‐MYO1D for 24, 48, or 72 h and subjected to immunoblot assay, MTT assay, or transwell invasion assay. The histograms of the cell proliferation and invasion analyses were obtained as in Figure 3A and 3D, respectively. [file CTM2-11-e515-s003.tif]

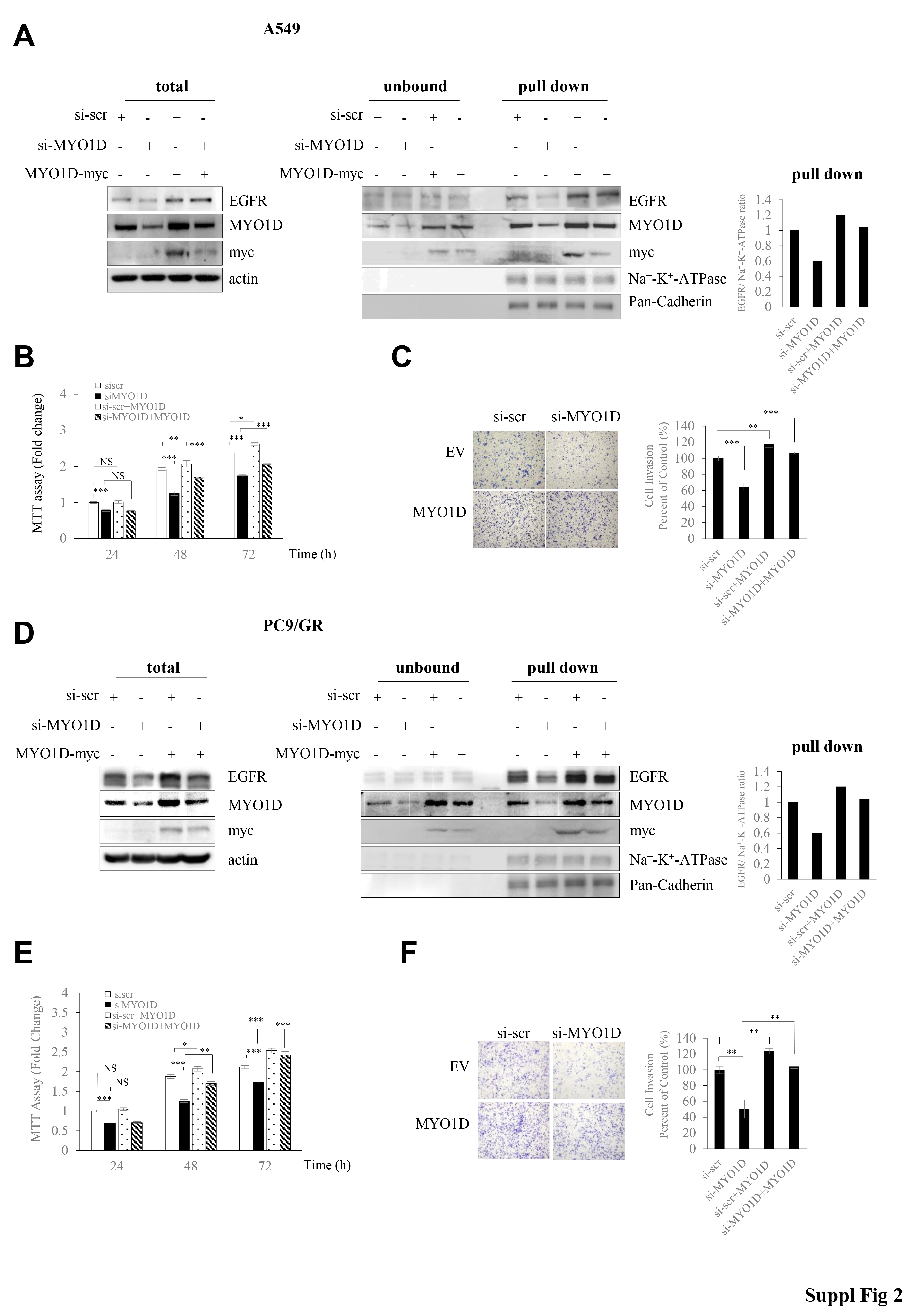

Supplement: Supplementary file 2 — FIGURE S2 Expression of exogenous MYO1D restores the decreases in the EGFR level, cell proliferation, and motility caused by treatment with MYO1D si‐RNA in NSCLC cells. Rescue experiments were performed by transfection with MYO1D siRNAs (siRNA#1 and siRNA#2) overnight followed by transfection of the plasmid, a construct expressing myc‐tagged MYO1D (MYO1D‐myc). A, Reduced EGFR level was rescued by the expression of exogenous MYO1D in the membrane fraction. MYO1D was overexpressed in A549 cells in the presence of siRNA targeting MYO1D. Plasma membrane preparations were obtained as in Figure 1E. B, Expression of exogenous MYO1D attenuated the suppression of cell proliferation by MYO1D depletion in A549 cells. C, Exogenous MYO1D protein restored the decrease in invasion capacity by MYO1D depletion in A549 cells. The histogram and pictures of the cell invasion analysis were obtained as in Figure 3D. D, Exogenous MYO1D protein rescued the EGFR level of PC9/GR cells in the membrane fraction after MYO1D depletion. E, The suppression of cell proliferation by MYO1D depletion was attenuated by exogenous MYO1D protein in PC9/GR cells. F, The decreased invasion capacity in PC9/GR cells caused by knockdown of MYO1D was restored by exogenous expression of MYO1D protein. [file CTM2-11-e515-s005.tif]

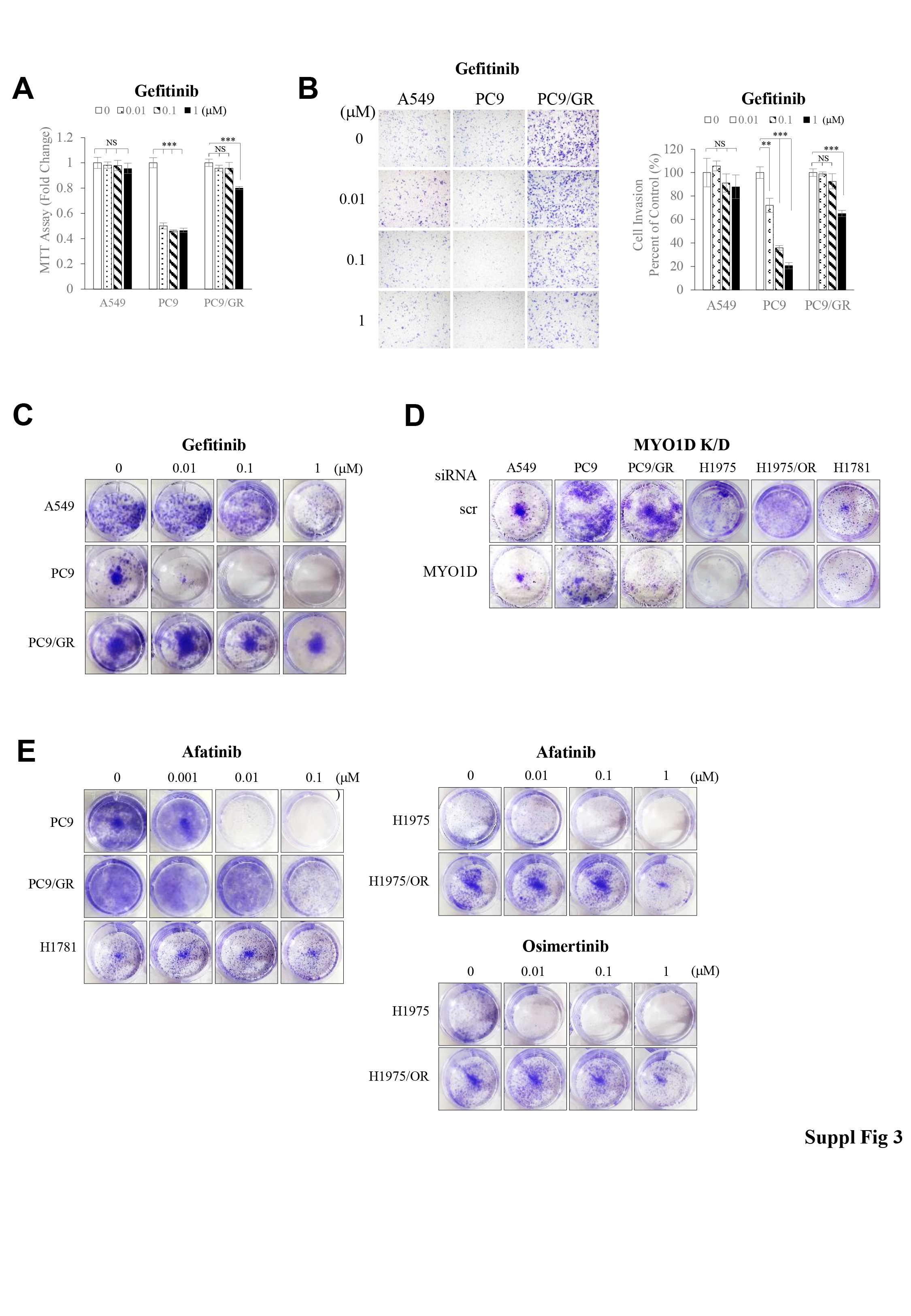

Supplement: Supplementary file 3 — FIGURE S3 Knockdown of MYO1D reduces long‐term cell survival in NSCLC cells expressing either wild‐type or mutant EGFR, which show varying sensitivity to TKIs. A, NSCLC cells expressing either wild‐type or mutant EGFR show varying sensitivity to gefitinib. A549, PC9, or PC/9GR cells were treated with gefitinib for 48 h and cell proliferation was determined by MTT assay. B, Effect of gefitinib on cell invasiveness in NSCLC cells expressing either wild‐type or mutant EGFR. The invasion capacity of cells after treatment with the indicated concentrations of gefitinib for 24 h was determined by using the transwell invasion assay. The histogram and pictures of the cell invasion analysis were obtained as in Figure 3D. C, Effect of gefitinib on survival of NSCLC cells. Long‐term cell survival was determined by clonogenic assay after treatment with gefitinib in A549, PC9, or PC9/GR cells. After incubation for 14 days, staining of attached cells was performed with trypan blue solution. D, Knockdown of MYO1D suppresses the survival of NSCLC cells. Long‐term cell survival was determined by clonogenic assay after MYO1D depletion. A549, PC9, PC9/GR, H1975, H1975/OR, or H1781 cells were transfected with either si‐scr or si‐MYO1D. After incubation for 14 days, staining of attached cells was performed with trypan blue solution. E, Effect of various TKIs on survival of NSCLC cells. Long‐term cell survival was determined by clonogenic assay after treatment with afatinib or osimertinib in A549, PC9, or PC9/GR, H1781, H1975, or H1975/OR cells. After incubation for 14 days, attached cells were stained with trypan blue solution. [file CTM2-11-e515-s006.tif]

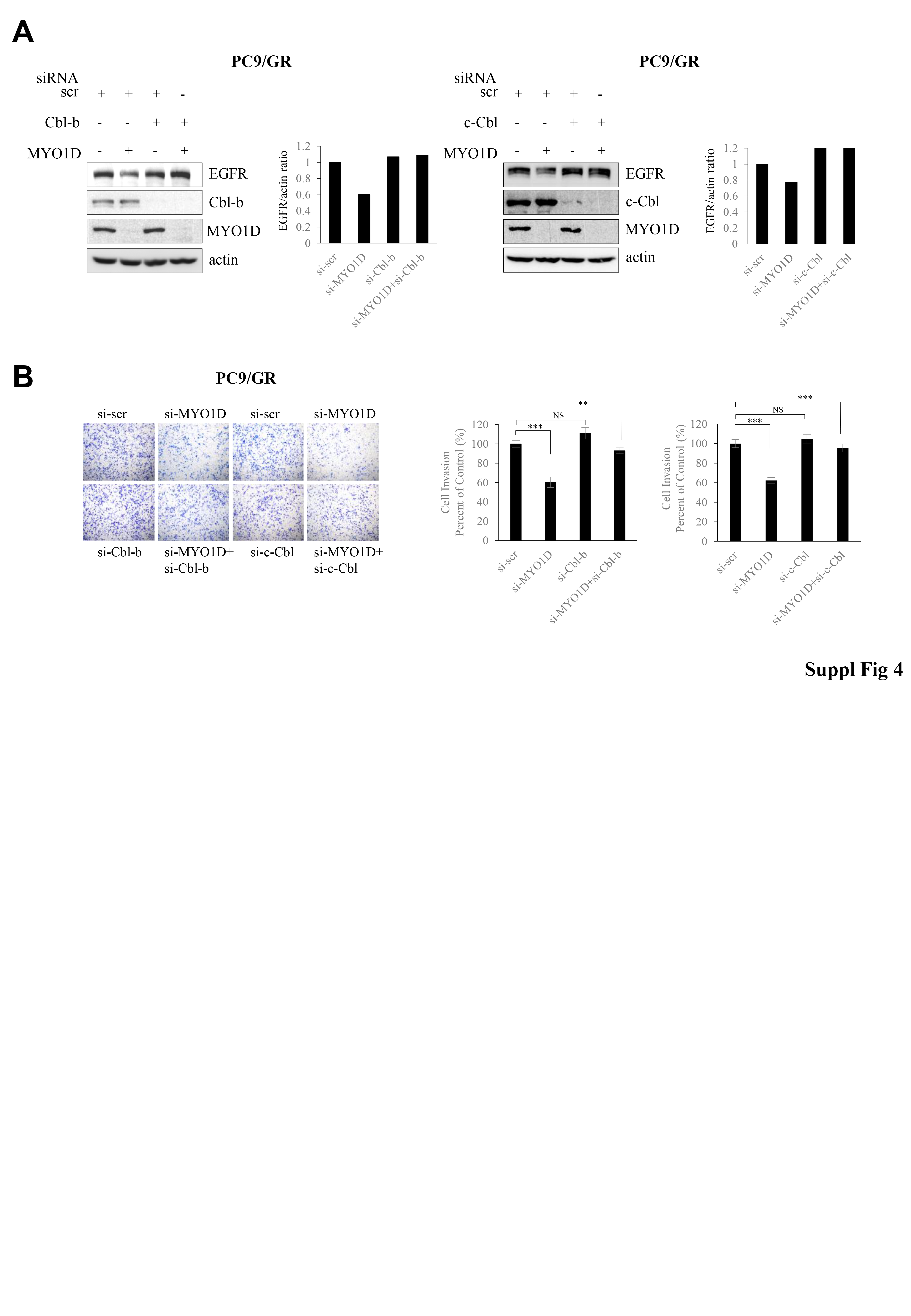

Supplement: Supplementary file 4 — FIGURE S4 Another set of specific si‐RNAs for Cbl‐b or c‐Cbl also restores the decreased EGFR level and invasion capacity in MYO1D‐depleted PC9/GR cells. A, Knockdown of Cbl‐b (left, mixed si‐Cbl‐b #1 with #2, see Materials and Methods) or c‐Cbl (right, mixed si‐c‐Cbl #1 with #2, see Materials and Methods) restored the reduced EGFR level in MYO1D‐depleted PC9/GR cells. After transfection, cells were immunoblotted with the indicated antibodies. B, Depletion of Cbl‐b or c‐Cbl restored the reduced invasion capacity in MYO1D‐depleted PC9/GR cells. The histogram and pictures of the cell invasion analysis were obtained as in Figure 3D. [file CTM2-11-e515-s002.tif]

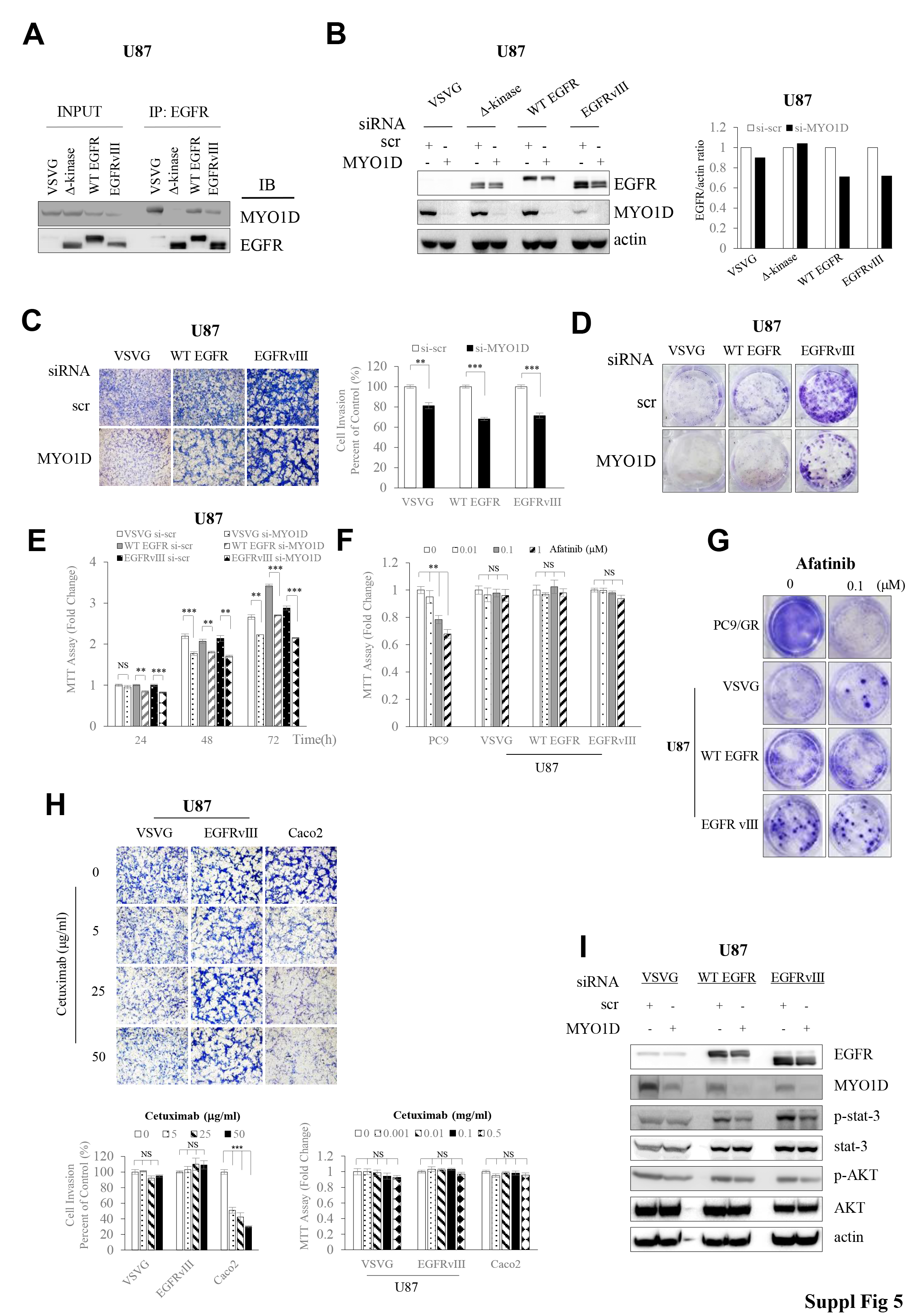

Supplement: Supplementary file 5 — FIGURE S5 MYO1D retains the wild‐type EGFR and mutant EGFRvIII in the plasma membrane of U87 glioblastoma cells. A, MYO1D interacts directly with endogenous mutant EGFRvIII and wild‐type EGFR. U87 glioblastoma cells were transfected with each deletion mutant of EGFR, such as empty vector (vsvg), kinase‐domain deleted EGFR construct (Δ‐kinase), or exon 2–7 deleted EGFR construct (EGFRvIII, in‐frame 801 nucleotides‐deleted in the extracellular domain). Each cell lysate was immunoprecipitated with an EGFR antibody and analyzed by immunoblotting with the indicated antibodies. B, Knockdown of MYO1D inhibits the expression level of wild‐type and mutant EGFRs in glioblastoma cells. U87 cells were cotransfected with each deletion mutant of EGFR and si‐scr or si‐MYO1D for 48 h. After transfection, cells were immunoblotted with the indicated antibodies. C, Depletion of MYO1D suppresses the invasion capacity of glioblastoma cells expressing wild‐type EGFR or mutant EGFRvIII. U87 cells transfected with either si‐scr or si‐MYO1D for 48 h were subjected to transwell invasion assay. The histogram of the invasion assay was obtained as in Figure 3D. D, Knockdown of MYO1D suppresses the survival of glioblastoma cells expressing wild‐type EGFR or mutant EGFRvIII. U87 cells were transfected with either si‐scr or si‐MYO1D and long‐term cell survival was determined by clonogenic assay as in Supporting Information Figure S3D. E, Knockdown of MYO1D suppresses proliferation in glioblastoma cells expressing wild‐type EGFR or mutant EGFRvIII. Cell proliferation was determined by MTT assays at 24, 48, and 72 h after cotransfection with each deletion mutant of EGFR and si‐scr or si‐MYO1D. F, The proliferation of glioblastoma cells was little affected by afatinib. PC9, PC/9GR, U87/wild‐type EGFR, or U87/EGFRvIII cells were treated with afatinib for 48 h and sensitivity was determined by MTT assay. G, The survival of glioblastoma cells was also little affected by afatinib. Long‐term cell survival [file CTM2-11-e515-s004.tif]
