# Supplementary material for: New strategy for suppressing the growth of lung cancer cells harboring mutations in the ATP‐binding region of EGFR by targeting the molecular motor MYO1D
Source: Clin Transl Med. 2021 Aug 6;11(8):e515. doi: 10.1002/ctm2.515 (PMC8343539; doi:10.1002/ctm2.515)
Supplement: Supplementary file 6 — Supporting Information [file CTM2-11-e515-s001.doc]

**1 MATERIALS AND METHODS**

**1.1 Cell culture**

Human NSCLC cell lines (A549, PC9, PC9/GR, HCC827, NCI-H1650, H1975, and H1781) were obtained from American Type Culture Collection. H1975/OR and U87-MG glioblastoma cells were kindly provided by Dr. Jin Kyung Rho (Asan Medical Center, Seoul, Korea) and Dr. Ho-Shin Gwak(National Cancer Center, Goyang, Korea), respectively. Cells were cultured in RPMI‐1640 medium, or DMEM containing 10% fetal bovine serum (GenDEPOT), 100 units/mL of penicillin and 100 µg/mL of streptomycin (Corning), and were incubated at 37°C in an atmosphere with 5% CO2.

**1.2 Antibodies and reagents**

Antibodies to EGFR, MYO1D, Na^+^-K^+^ ATPase, total ERK, and Ubiquitin (Santa Cruz Biotech); phospho-EGFR (Y1068), ErbB2, Cbl-b, c-Cbl, GAPDH, AKT, p-AKT, p-ERK, p-p38, p38, p-stat-3, and stat-3 (Cell Signaling Technol); Myc (MBL); HA and Actin (Sigma) were used with appropriate secondary antibodies (MBL). Gefitinib, afatinib, andosimertinib were purchased from Selleck Chemicals.

Human recombinant EGF (R&D systems) were used at concentrations of 100 ng/ml. For treatment experiments, cells were first serum-starved overnight and then stimulated with the EGF.

**1.3 Western blot and immunoprecipitation**

NSCLC cells or glioblastomas cells were harvested and lysed. Lysates were separated, transferred, and immunoblotted as previously described.^12^ The blot was reprobed with anti-actin antibody or anti-GAPDH antibody to control for loading. Cell lysates from NSCLC or glioblastoma cells were used for immunoprecipitation experiments as previously described.^13^

**1.4 RNA interference and plasmids**

For the siRNA interference experiments, we purchased siRNAs specific to human MYO1D (Invitrogen and Santa Cruz Biotech), Cbl-b (Invitrogen and Santa Cruz Biotech), or c-Cbl (Invitrogen and Santa Cruz Biotech). Sequences were as follows; si-MYO1D (#1) 5'-GCGCCUUAUGUAUAACAGUUCAAAU-3'; si-MYO1D (#2) 5'-CCCUUACAAGUUGUUGAACAUCUAU-3';si-MYO1D (#3 **is a pool of 3 different siRNAs**) 5'-CUCUCUACAUCUCCAGAAATT-3', 5'-CAGAAUUGCUCUCUACUA

ATT-3', 5'- CCAAGGAUCAUCUUACUUATT-3';Cbl-b (#1) 5'-UCAUCCCACCCU

GUUUCCCUGAAUU-3’;Cbl-b (#2 **is a pool of 3 different siRNAs**) 5'-GCAAGGAG

AGAAUGUAUGAtt-3',5'-CUCGCCUGGAUCUAAUUCAtt-3',5'-CCAGUUCCAU

UUCUCUCAUtt-3'; c-Cbl (#1) 5'-CCAGCAGAUUGAU-AGCUGUACGUAU-3’; c-Cbl (#2 **is a pool of 4 different siRNAs**) 5'-GACACAUUUCGGAUUACUATT-3', 5'-CUCUGAAAUCCACUAUUGATT-3', 5'-GACGUAUGACGAAGUGAAATT-3', 5'-CCACAUUCCAACUAUGUAATT-3'. For the nonspecific scrambled siRNA (si-scr), All Stars Negative Control siRNA (Qiagen) was used.Transfection of the siRNA duplexes was performed according to a protocol provided by Lipofectamine^TM^RNAiMAX. Cells were treated for 48 h to allow for maximum knockdown, and then used for RT-PCR and Western blot analysis or invasion assays. The efficiency of usual knockdown was 70% to 80% as analyzed by Q-PCR results of target mRNA after si-RNA transfection.

Expression constructs were generated by PCR-based methods: Myc-tagged MYO1D, HA-tagged EGFR, HA-tagged EGFR deletion mutants. All constructs were confirmed by sequencing.

**1.5 Cell invasion assay**

Transwell cell migration assays were performed as previously described.^12^ Briefly, cultured cells pretreated with siRNA (for 48 h), gefitinib, afatinib, or osimertinib were plated into the top of a 24-well invasion chamber assay plate (Costar). Conditioned DMEM medium containing 10 μg/ml of fibronectin (Calbiochem) or 10% FBS (H1781) was added to the bottom chamber as a chemoattractant. After 24 h of incubation, the cells were stained. Cells at the top surface of the upper chambers were wiped off with a cotton ball, and migrated cells on the bottom surface were counted in five random squares of 0.5 mm × 0.5 mm for each upper chamber. The results are shown by the mean ± SEM of the number of cells per field.

**1.6 Clonogenic survival assays**

Clonogenic survival assays were determined with cells treated in the presence of TKI or si-MYO1D RNA. Cultures were grown for 14 days, fixed with acetic acid–methanol (1:3 V/V), and then stained with 0.25% crystal violet in 80% methanol.

**1.7 Subcellular fractionation**

Cytoplasmic and membrane fractions were prepared using the manufacturer’s instructions for a subcellular protein fractionation kit (Thermo Scientific). Each fraction was resolved by SDS-PAGE and probed for EGFR and MYO1D. Fraction purity was assessed by probing for Na^+^-K^+^ ATPase or Pan-Cadherin.

**1.8 Cell surface protein isolation**

The Pierce Cell Surface Protein Isolation Kit (Thermo Scientific) was used for the isolation and collection of surface proteins.^14^ In brief, cultured cells were incubated with Sulfo-NHS-SS-Biotin at 4 °C for 30 min, followed by quenching the biotinylation reaction. The cell lysates were prepared using the provided lysis buffer in the presence of a protease inhibitor cocktail (GenDEPOT). To capture biotinylated (surface) proteins, lysates in a column were incubated with NeutrAvidin Agarose gel for 2 h. The unbound (unbiotinylated) proteins, which represent intracellular fraction, were separated by centrifugation from the captured surface proteins in the column. The intracellular fraction was stored at −20 °C to use as an internal control for the process of surface protein isolation. Finally, the captured surface proteins were eluted with dithiothreitol/PBS from the biotin-NeutrAvidin Agarose. The eluted cell surface proteins were collected by column centrifugation.

**1.9 Cell viability assay**

Cell viability was determined in a 3-(4,5-Dimethylthiazol-2-yl)-2,5-diphenyltetrazolium bromide (MTT) assay by using the EZ-Cytox cell viability assay kit (Daeil Lab Service Co., Korea). Briefly, cells were cultured in 96-well plates (5 × 10^3^ cells/well). The culture medium was removed after 24, 48 or 72 h. Next, 10 μl of EZ-Cytox reagent added and incubated at 37°C for another 2 h prior to measurement. The absorbance was measured in a micro-plate spectrophotometer at 450 nm.

**1.10 Confocal Microscopy**

A594, PC9 or PC9/GR cells were seeded on fibronectin-coated slides (Becton Dickinson), and si-scr or si-MYO1D was transfected for 48 h. After fixation, monolayered cells were double-labeled with EGFR and MYO1D antibodies and visualized with Alexa 488- and 568-conjugated secondary antibodies (Thermo Fisher), respectively. DAPI was used for nuclear counter staining.**All images were obtained with a Laser Scanning Confocal Microscope (Leica TCS SP5/AOBS**, Germany**) at Gwangju Center in Korea Basic Science Institute.**

**1.11 TIRF Microscopy**

A594 or PC9/GR cells were cultured in 35-mm culture dishes with no. 1.5 glass bottoms (Greiner Bio One GmbH, Germany) for TIRF microscopy. EGFR and MYO1D proteins were labeled with Alexa 488- and 568-conjugated secondary antibodies (Thermo Fisher) after fixation. TIRF images were obtained using the Elyra Super Resolution Microscope System (Carl Zeiss Microscopy, Germany**) at Gwangju Center of Korea Basic Science Institute.** Double labeled proteins were sequentially excited with 488 and 561 nm laser lines and imaged with a 100× oil objective (NA 1.46) and a back-thinned EMCCD camera (AndoriXON 897, UK). To quantify the expression level of EGFR and MYO1D proteins, Imaris software was used to count fluorescently labeled spots on TIRF images, and co-localization of both proteins was determined and counted with the Coloc module in the same software package (Bitplane, Switzerland).

**1.12 Statistical analysis**

Statistical significances were measured between the experimental groups by using ANOVA followed by Tukey HSD post hoc test or Student’s *t* test. All statistical analyses were two-sided, and *P* values less than 0.05 were considered as statistically significant. Analyses were performed with PASW Statistics 20 (SPSS, an IBM Company, Chicago, IL) software.

**1.13 References**

12. Lee JH, Park SR, Chay KO, et al. KAI1 COOH-terminal interacting tetraspanin (KITENIN), a member of the tetraspanin family, interacts with KAI1, a tumor metastasis suppressor, and enhances metastasis of cancer. *Cancer Res*. 2004;64:4235-4243.

13. Kho DH, Bae JA, Lee JH, et al. KITENIN recruits Dishevelled/PKC delta to form a functional complex and controls the migration and invasiveness of colorectal cancer cells. *Gut.* 2009;58:509-519.

14. de Wit M, Jimenez CR, Carvalho B, et al. Cell surface proteomics identifies glucose transporter type 1 and prion protein as candidate biomarkers for colorectal adenoma-to-carcinoma progression. *Gut.* 2012;61:855-864.
